# Antioxidant gene expression analysis and evaluation of total phenol content and oxygen-scavenging system in tea accessions under normal and drought stress conditions

**DOI:** 10.1186/s12870-021-03275-0

**Published:** 2021-10-27

**Authors:** Mehdi Rahimi, Mojtaba Kordrostami, Fereshteh Mohamadhasani, Sanam Safaei Chaeikar

**Affiliations:** 1 Department of Biotechnology, Institute of Science and High Technology and Environmental Sciences, Graduate University of Advanced Technology, Kerman, Iran; 2 Department of Plant Breeding, Nuclear Agriculture Research School, Nuclear Science and Technology Research Institute (NSTRI), Karaj, Iran; 3 Biology Department, Faculty of Science, Payame Noor University (Pnu), Bam, Iran; 4 Tea Research Center, Horticultural Sciences Research Institute, Agricultural Research, Education and Extension Organization (AREEO), Lahijan, Iran

**Keywords:** Drought stress, DPPH-free radicals assay, IC50, Phenol content, Gene expression

## Abstract

**Background:**

Abiotic and biotic stresses induce oxidative processes in plant cells that this process starts with the production of ROSs which cause damage to the proteins. Therefore, plants have increased their antioxidant activity to defend against this oxidative stress to be able to handle stress better. In this research, 14 different tea accessions in a randomized complete block design with two replications were evaluated in two normal and drought stress conditions, and their antioxidant activity was measured by DPPH-free radicals’ assay and gene expression analysis.

**Results:**

The results of gene expression analysis showed that the 100 and 399 accessions and Bazri cultivar had high values for most of the antioxidant enzymes, ascorbate peroxidase, superoxide dismutase, catalase, and peroxidase under drought stress conditions while the 278 and 276 accessions had the lowest amount of antioxidant enzymes in the same situation. Results showed that the IC50 of the BHT combination was 90.12 μg/ ml. Also, The IC50 of accessions ranged from 218 to 261 μg/ml and 201–264 μg/ml at normal and drought stress conditions, respectively. The 100 and 399 accessions showed the lowest IC50 under normal and drought stress conditions, while 278 and 276 accessions had the highest value for IC50. The antioxidant activity of tea accession extracts under normal conditions was ranged from 25 to 69% for accessions 278 and 100, respectively. While, the antioxidant activities of extracts under drought stress condition was 12 to 83% for accessions 276 and 100, respectively. So, according to the results, 100 and 399 accessions exhibited the least IC50 and more antioxidant activity under drought stress conditions and were identified as stress-tolerant accessions. However, 278 and 276 accessions did not show much antioxidant activity and were recognized as sensitive accessions under drought stress conditions.

**Conclusions:**

These results demonstrate that total phenol content, antioxidant activity, and the oxygen-scavenging system can be used as a descriptor for identifying drought-tolerant accessions.

## Background

Tea (*Camellia sinensis* L.) is an aromatic drink that is native to Asian countries and is usually made by pouring hot or boiling water on its leaves. Tea removable product is an umbilical shoot, which is harvested once, every 7 to 12 days, depending on the environmental conditions and type of cultivars. Tea bushes in a garden are not only different regarding morphological, physiological, and yield-related traits, but the quality of each plant is different from another [1]. Drought avoidance in the tea is due to morphological changes such as decreasing stomatal conductance, decreasing leaf area, and developing in the root system. On the other hand, drought tolerance is induced by physiological and molecular mechanisms including photosynthesis, osmotic regulation, antioxidant production, and scavenging of reactive oxygen species (ROS) [2].

Most natural environments contain biotic and abiotic stresses for plants [3, 4]. Drought and salinity as two abiotic stressors in the process of desertification have a significant impact on limiting crop growth and reducing crop production [5, 6]. Therefore, a detailed understanding of the mechanisms involved in coping with these stresses in plants will greatly improve the plants’ resistance [7]. Among the environmental factors, drought is the main factor limiting the growth of plants [8]. Drought is the most common environmental stress, which has almost limited production to about 25% of the world’s arable lands [9]. So, the distribution of plants around the world is largely affected by the amount of water [10]. As a result, among the important stressors, drought is the second major cause of yield loss after pathogens [11]. One of the first reactions to water stress is a decline in growth [12]. In general, plants have evolved through morphological and physiological strategies to tolerate drought stress [13]. Due to the limited water resources, the identification and planting of drought-tolerant plants with high yield potential are of great importance [3].

Under normal conditions, low levels of reactive oxygen species (ROS) such as superoxide radicals, hydrogen peroxide, and hydroxyl radicals, are part of the metabolites produced by plant cells [14]. When the balance between the different parts of a cell is severely disturbed by the production and elimination of ROS, oxidative stress and its damage occur [15]. Different abiotic stresses in the plants lead to excessive production of reactive oxygen species (ROS) and cause damage to the proteins, lipids, carbohydrates, and DNA. Drought stress causes water deficiency in plant tissues resulting in osmotic effects on a wide range of plant metabolic activities. Reactive oxygen species, which are due to ionic stress and hyperosmotic effects, cause membrane defects and cell death [16]. Most of the damage to plants exposed to drought is related to oxidative damage at the cellular level [17]. To counteract the oxidative stress in plants, the antioxidant defense system such as catalase and ascorbate peroxidase acts to protect plants. Enzymatic antioxidant defense systems in plants have been developed to reduce the oxidative damage caused by the ROS [18]. Drought stress leads to oxidative stress, which causes the accumulation of ROS mainly in the chloroplast and mitochondria. Most of the ROS molecules include hydroxyl radicals, superoxide anion radicals, singlet oxygen, and hydrogen peroxide. Drought stress in the plants activates some of the defense mechanisms to protect against the harmful effects of oxidative stress. Increasing the antioxidant activity in the plants will cause a better tolerance and resistance against oxidative damage [19]. The ROS reduction mechanism is one of the defensive mechanisms against abiotic stress. Plants naturally produce different types of antioxidants to reduce the toxicity of ROS, which results from drought tolerance and reduced oxidative damage [20]. The reduction of ROS is done by antioxidant enzymes such as peroxidase, superoxide dismutase, and catalase [21, 22].

Reports indicate that exposure to a variety of environmental stresses, including drought, can exacerbate ROS production, leading to increased oxidation of cellular components and oxidative stress in plants [23–25]. Antioxidant enzymes are highly sensitive to environmental stressors and act as markers of increasing or decreasing gene expression in plants for water deficit conditions [26]. Mechanisms of plant survival under stress conditions depend on different responses, including plant capacity to maintain high antioxidant levels, whether enzymatic or non-enzymatic [27]. In a study, the activity of superoxide dismutase and FeSOD isoforms increased water use efficiency (WUE) in potato genotypes under water deficit conditions [28]. In another study, an increase in catalase and ascorbate peroxidase was observed in canola (*Brassica napus*) under water-deficient conditions [29].

In recent decades, significant improvements in drought tolerance of crop varieties have been made through conventional selection and breeding methods. However, many selection methods have been based on differences in agronomic characteristics. Agronomic traits represent a combination of genetic and environmental effects on plant growth and incorporate physiological mechanisms to confer drought tolerance. Common crop selection parameters for drought tolerance include yield, survival, plant height, leaf area, leaf damage, relative growth rate, and relative growth decline. When screening for components of complex traits is performed, physiological and biochemical criteria can provide more information than agronomic or visual evaluation parameters [30]. On the other hand, to avoid reducing the product due to water stress, it is necessary to carefully select the appropriate cultivars for planting in the fields. Accession cultivars (modified cultivars based on non-sexual propagation) with less water requirement significantly increase the plant’s ability to withstand drought stress and associated stresses (such as oxidative stress).

In the study of M Rahimi, M Kordrostami and M Mortezavi [31] based on biochemical and morphological traits and also in the other study [32] based on drought tolerance indices and multivariate analysis, the accessions used in this study were evaluated in both normal and drought conditions and 100, and 399 accessions and Bazri cultivar were identified as the tolerant genotypes and 278, 276 and 285 accessions were identified as the sensitive ones. Phenolic compounds are the most widely used secondary metabolites in plants. They belong to a large group of non-nutritious and biologically active compounds [33]. Considering that the more total phenol content or antioxidant activity of plants under drought conditions leads to better drought tolerance of the plant, thus, this study was performed to identify tea tolerant drought accessions based on total phenol content, total antioxidant activity, or free radical scavenging activity. In addition, by comparing tolerant and sensitive accessions based on total phenol content or antioxidant activity with tolerant and sensitive accessions based on previous studies [31, 32], total phenol content, antioxidant activity, and the oxygen-scavenging system can be investigated as an indicator to identify tolerant and sensitive accessions.

## Results

The results of simple analysis of variance (ANOVA) showed that there was a significant difference between tea accessions in terms of % inhibition of DPPH activity, IC50, and total phenolic compounds in both drought stress and non-stress conditions (Table 1). Also, the mean comparison for % inhibition of DPPH activity, IC50, and total phenolic compounds in both normal and stress conditions is shown in Table 2. The mean comparison showed that the 100 accession had the highest % inhibition of DPPH activity and the 278 accession had the least % inhibition of DPPH activity in both conditions (Table 2).

**Table 1:** Simple ANOVA for IC50, Inhibition, and total phenol content (%) traits under normal and drought stress conditions

S.O.V	df	Mean squares (MS)					
		Normal conditions			Drought Stress conditions		
		IC50	Inhibition (%)	Total phenol content	IC50	Inhibition (%)	Total phenol content
Block	1	0.0496^ns^	0.00013^ns^	0.0057^ns^	0.3566^ns^	0.0892^ns^	0.0032^ns^
Accessions	13	334.4899**	402.1953**	1.6037**	961.87132**	1144.2049**	6.9874**
Error	13	0.6897	0.0224	0.0042	0.8937	0.0498	0.0139
CV (%)		0.35	0.31	0.85	0.41	0.48	1.53

^ns^ and ^**^: Non-significant and significant at the probability level of 1%, respectively

**Table 2:** Mean comparison of Inhibition (%), IC50, and total phenol content under normal and drought stress conditions

Accessions	Normal conditions			Drought Stress conditions		
	Inhibition (%)	IC50	Total phenol content	Inhibition (%)	IC50	Total phenol content
74	58.18d	225.89i	8.35c	68.21d	210.67j	9.3d
100	69.32a	218.25 k	8.85a	83.24a	201.32 l	10.6a
252	39.60j	241.49e	7.15 h	29.48j	247.64d	6.55i
261	42.47i	236.62f	7.35 g	34.40i	242.31e	7 h
269	45.25 h	234.44f	7.75f	40.39 h	236.52f	7.75 g
270	49.37 g	231.20 g	7.95e	48.51 g	229.61 g	8.2f
272	35.23 k	246.03d	6.85i	25.31 k	250.14d	6.2i
276	28.60 m	255.45b	6.35 k	15.52 m	260.21b	5.3 k
277	55.38e	227.55ih	8.25 cd	62.23e	214.31i	8.6e
278	25.17n	258.32a	6.15 l	12.38n	264.12a	4.75 l
280	52.49f	228.52 h	8.1ed	54.50f	223.42 h	8.35ef
285	32.36 l	251.26c	6.65j	19.75 l	254.32c	5.7j
399	67.24b	222j	8.7ab	76.24b	203.58 l	10.2b
bazri	62.18c	223.01j	8.55b	72.19c	206.89 k	9.75c

Treatments with the same alphabets are not statistically different at the 1% probability level

The IC50 values of methanolic extracts of tea accessions were shown under normal and drought stress conditions. Tea accessions showed different IC50 under normal and drought stress conditions. The 100 and 399 accessions showed the lowest IC50 under normal and drought conditions, while 278 and 276 accessions had the highest value for IC50. The value of IC50’s of other accessions was between these values. Under drought stress conditions, the IC50 value for accessions 100 and 399 was lower than other accessions. Also, the IC50 value of these accessions was lower than the normal conditions. While 278 and 276 accessions showed the highest value for IC50 under drought stress conditions and the IC50 value of these accessions was less than normal conditions. The IC50 of BHT combination, an antioxidant used in the food industry, was 90.12 μg/ml in this study.

The IC50 of accessions ranged from 218 to 261 μg/ml and 201–264 μg/ml under normal and drought stress conditions, respectively. According to the preliminary experiments, concentrations of 250 μg/ml were selected for the evaluation of % inhibition of DPPH activity under both conditions. The existence of a significant difference between accessions in both experiments can indicate a high genetic diversity in the studied populations in terms of % inhibition of DPPH activity (Table 1). The antioxidant activity of tea accession extracts under normal conditions was ranged from 25 to 69% for 278 and 100 accessions, respectively. While, the antioxidant activities of extracts under drought stress conditions was ranged from 12 to 83% for 276 and 100 accessions, respectively. In the present study, the mean square of the total phenolics of 14 tea accessions showed significant differences in both normal and drought stress conditions (Table 2). Among the 14 studied accessions, the 100 and 399 accessions had a higher phenolic content in both conditions, compared to the other tea accessions (Table 2).

**Fig. 1:**
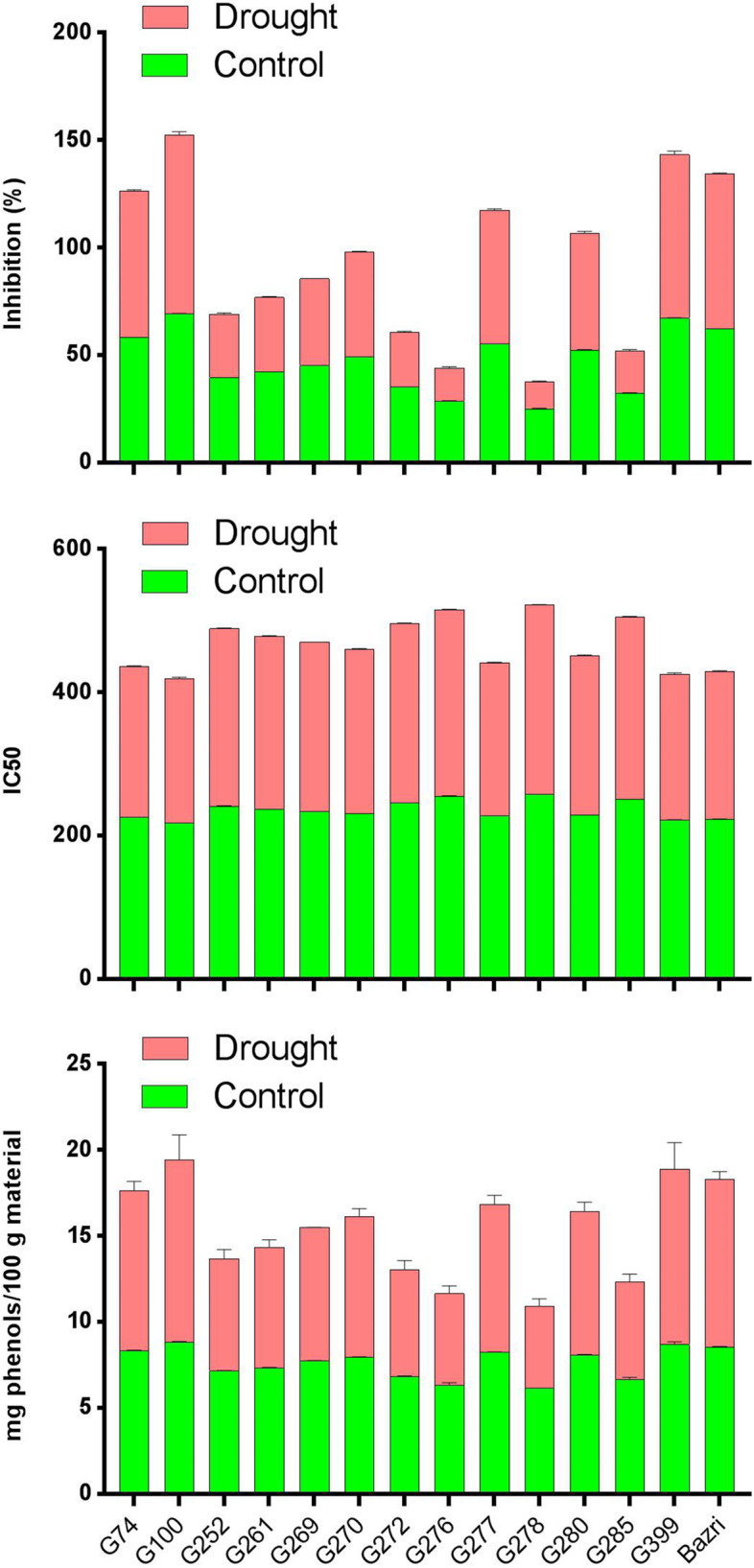
Mean comparison for inhibition (%), IC50, and total phenol content. Treatments with the same letters are not statistically different

To make a significant difference between the accessions, make the calculations more accurate and not attributed to the genotype × environment interaction; the combined analysis of variance for two experiments was carried out in the form of a pooled analysis. The results showed that total phenol content in all the accessions showed a different behavior after irrigation. In the 278 and 276 accessions, the total phenol content decreased compared to the normal conditions, while the total phenol content increased for the 100 and 399 accessions in the same situation. The results of combined analysis of variance for % inhibition of DPPH activity showed that the environmental effect was significant at 1% probability level (Table 3), in the sense that the normal and drought stress environment did not have the same effect on the trait, or, in other words, the change in the amount of this trait was significant in the two humidity conditions. Also, there was a significant difference between the studied accessions for % inhibition of DPPH activity (Table 3). The accession × stress interaction effect was also significant that indicate the reaction of accessions was not the same under different moisture conditions. Considering the significant effect of accession× stress interaction, the mean comparison showed that the 100 accession had the highest % inhibition of DPPH activity under the stress conditions and then the 399 accession was ranked next. While, the 276 and 278 accessions had the lowest % inhibition of DPPH activity under the stress conditions, respectively (Fig. 1). According to the results, it was observed that the accessions that have been identified as the tolerant and sensitive accessions based on the total phenol content, antioxidant activity, and the oxygen-scavenging system in this study, have been identified as tolerant and sensitive accessions in previous studies and the results of this study confirm the results of previous studies. Therefore, these traits can be used as indicators to identify tolerant and sensitive accessions.

**Table 3:** Combined analysis of variance for IC50, inhibition (%), and total phenol content

S.O.V	Df	MS of Traits		
		Inhibition (%)	IC50	Total Phenol content
Stress	1	29.98858^b^	215.84363^b^	0.1116^a^
E_1_	2	0.04464	0.20309	0.0045
Accession	13	1447.92487^b^	1199.24601^b^	7.6172 ^b^
Stress×Accession	13	98.47536^b^	97.11520^b^	0.9739 ^b^
Error	26	0.03606	0.79167	0.0091
CV (%)		0.41	0.38	1.24

^a^ and ^b^: Significant at the probability level of 5 and 1%, respectively

Also, because antioxidants enzymes change under drought stress conditions, accessions with high levels of these enzymes were more tolerant and less damaged under drought stress. Therefore, the expression of the genes encoding these enzymes was examined to compare with the results of the oxygen-scavenging system in this study and previous studies [31, 32].

**Fig. 2:**
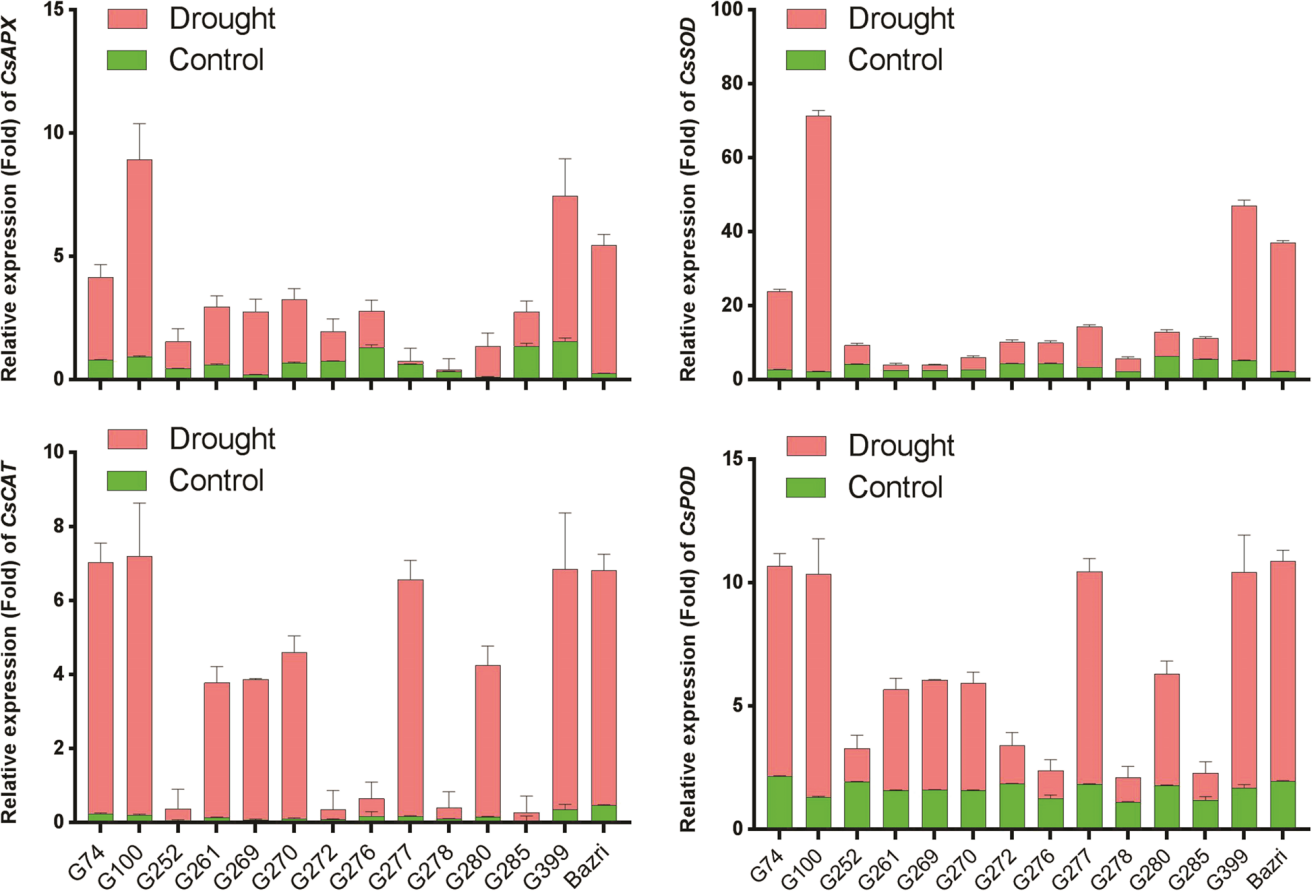
qPCR analysis of the expression profiles for the antioxidant genes during drought stress in the tea accessions. The error bars represent standard deviation among the biological replicates

To ensure the quality, the extracted RNA was quantitatively and qualitatively evaluated by spectrophotometer and gel electrophoresis. The presence of two bands, 28S and 18S, indicated excellent RNA quality. The results of gene expression analysis showed that the 100 and 399 accessions and Bazri cultivar had high values for most of the antioxidant enzymes, ascorbate peroxidase, superoxide dismutase, catalase, and peroxidase under drought stress conditions while the 278 and 276 accessions had the lowest amount of antioxidant enzymes in the same situation (Fig. 2).

The results of antioxidant gene expression also showed that accessions that were identified as tolerant and sensitive accessions based on biochemical traits were also identified as tolerant and sensitive accessions based on the gene expression and confirmed the previous results [31, 32].

## Discussion

The results showed that drought-tolerant accessions have higher total phenol content, antioxidant activity, and a stronger oxygen-scavenging system. Previous studies have shown higher phenol content in the tolerant genotypes of tea [34] and other plants [35–38] under drought stress conditions. Therefore, these traits can be used as indicators to identify drought tolerant and sensitive genotypes [33]. The leaf is the source of photoassimilates, which yield the precursors of secondary metabolites such as malonyl-CoA and p-coumaroyl-CoA [39] which are light-dependent. In resource-limited environments, the theory of functional balance explains why shoot growth and not leaf growth correlate with phenolic content [40]. This provides pointers, that although total tea polyphenol content may be related to water-stress tolerance in tea, flavonoid derivatives, particularly the rich tea catechins might be more useful and should be the subject of further investigations. Besides absolute amounts of polyphenols, the results of this study showed varied fluctuation of polyphenol content and suggest that clones with more stable polyphenols are more tolerant to drought stress (Table 2 and Fig. 1).

Now ABTS and DPPH free radicals have been investigated for screening plant samples to stress tolerance. The large increase in the free radical scavenging capacity of ABTS and DPPH has been associated with the degree of stress tolerance in plants [41]. The evaluation of drought tolerance as growth retardation and cell membrane stability has been investigated. These indices are distinguished among plant genotypes and are associated with enzymatic and non-enzymatic antioxidants [42]. Drought-tolerant plants generally have high antioxidant system capacity that increases against control plants compared to sensitive plants in response to stress conditions [43]. These traits were considered important physiological parameters of drought tolerance. The data showed that the ROS scavenging system is an essential part of the protective mechanism against drought stress in plant cells.

Plants are equipped with an antioxidant defense system against the damaging effects of reactive oxygen species which consists of two groups of enzymatic (ascorbate peroxidase, catalase, superoxide dismutase, glutathione reductase, guaiac peroxidase, and others) and non-enzymatic antioxidants (carotenoids, ascorbates, glutathione, vitamin E, and others) [44]. The metabolism of antioxidants is one of the pathways of plant metabolism that can affect abiotic stress tolerance. The changes and the number of antioxidant enzymes show a significant difference between plant species. At present, numerous researchers have shown that stress tolerance in plants is correlated with the activity of antioxidant enzymes and is proposed as an important mechanism of drought stress tolerance [45]. Rapid accumulation and maintaining a high activity of antioxidant enzymes can be considered as a better protection mechanism under drought stress in plant cells. The correlation between the degree of drought tolerance and the activity of the antioxidant enzyme in several plant species has been shown [46]. The results of measurement of antioxidant capacity showed that irrigation stop has changed the plant’s defense ability against drought-induced oxidative stress and accessions have shown different behavior. In a study, it has been shown that with the gradual increase of drought stress, the antioxidant activity of the plant decreases, hydrogen peroxide production increases, followed by injuries to the membranes and increased lipid peroxidation [47]. Peroxidation of membrane lipids, including chloroplast and mitochondria membranes, eliminates their selective permeability, and their physiological role reduces crop yields during drought stress [48].

The results of gene expression patterns showed that drought stress increased total antioxidant enzyme activity in all the accessions, which was significantly higher in the tolerant accessions than susceptible ones. Today, some researchers believe that increasing levels of antioxidants increase plant tolerance to environmental stresses [49–53]. It has been suggested that the scavenging of plant-free radicals is an important component of the stress protection mechanism [54] and that the activity of the antioxidant enzyme is highly correlated with osmotic resistance [16]. The cooperation of these components together creates very important cycles [15]. Implementation of these cycles as defense mechanisms enables the cell to prevent the production of reactive oxygen species (ROS) or to collect them and reduce their harmful effects [55]. In addition, the performance of these cycles increases the cellular redox potential and decreases the damage to critical biomolecules, making the cell in a more desirable state under stress conditions [5]. In general, drought adaptation depends on keeping the amounts of ROS relatively low by the antioxidant system [31]. These results are in line with the results of the current study. In this study, the tolerant accessions showed higher antioxidant activity.

Polyphenols are also a kind of non-enzymatic antioxidants that inactivate free radicals of lipids or prevent them from converting to free radicals to reduce stress [56]. The results of this study showed that the total phenolic compounds, depending on the accession and the degree of stress, have shown different variations. Since the amount of phenolic compounds in the plant is influenced by the factors such as genotype, environmental conditions, plant tissue type, and soil type [57], it is expected that this difference is related to plant capability (accession type) for the synthesis of these compounds in adverse physiological conditions. As the results show that the amount of tea polyphenols shoot was influenced by soil water content and its fluctuating [34]. Also, in the accessions where the number of polyphenols is more stable during stress or more polyphenols, they are more resistant to drought stress.

The ability of methanolic extracts of different tea accessions under normal and drought stress conditions on scavenging activity of DPPH free radicals showed that increasing the concentration of the extract also increased the ability of free radicals scavenging [58]. Differences in the IC50 value can be attributed to various factors such as differences in weather conditions, soil, altitude, extraction method, and solvent type [59].

Oxygen for plants, like other aerobic organisms, is like a double-edged blade. Although oxygen is essential to growth and development, continuous contact with it can lead to cell damage and ultimately cause cell death [60]. Abiotic and biotic stress induces oxidative processes in plant cells that this process starts with the production of ROSs which results from the inappropriate activity of the electric Carter transmission [61]. This is because oxygen in the molecule form is reduced to various forms of ROS, especially in free radical anions superoxide (O_2_^−^) and hydrogen peroxide (H_2_O_2_) forms, which themselves react with various cellular compositions, cause severe or irreparable damage, eventually result in cell death. ROSs in plant cells are produced by both induction and construction methods, but under normal conditions, the balance of cellular redox is maintained through a construction method that has evolved in a wide range of antioxidant mechanisms to destroy ROSs [62].

Various environmental stresses and internal stimuli were caused to disruption of the redox balance by increasing the ROS production or reducing antioxidant activity, which results in oxidative stress. In response to the increase in the amount of ROS, the expression of the encoding genes of antioxidant proteins is reduced in sensitive plants. In such a situation, the expression of the encoding genes involved in a wide range of cellular processes is also reduced. In addition, ROS has a series of message roles, other than roles that act under oxidative stress conditions. In contrast, in some plants, the expression of the encoding gene of antioxidant protein increases or does not change, which leads to tolerance to environmental stresses in plants [63].

Plants that have a strong antioxidant system under drought stress conditions can tolerate drought stress better. Therefore, these plants are known as stress-tolerant plants [64]. Previous studies have shown that different tea accessions and varieties differ in the total antioxidant activity or free radical scavenging activity in different environmental conditions and harvesting times [65]. Also, in a study conducted on Iranian accessions under normal conditions, the 100 accession, as in this study, had the higher total antioxidant activity or free radical scavenging activity [66].

## Conclusions

According to the results of this study, under drought stress conditions, the 100 and 399 accessions exhibited more antioxidant activity than normal conditions and they were better able to tolerate drought stress and were identified as stress-tolerant accessions. Also, sensitive and tolerant accessions were recognized by biochemical traits and their comparison [31] was the same as the results of this study, and therefore these methods act to identify sensitive and tolerant accessions. However, the 278 and 276 accessions did not show much antioxidant activity under drought stress conditions, and the antioxidant activity of these accessions was lower than normal conditions and was recognized as sensitive accessions.

## Material and methods

### Plant material

Since no experiments have been conducted to screen tea accessions for drought tolerance, 14 tea accessions (Table 4) were evaluated in a completely randomized block design with two separate replications under normal and drought stress conditions at the Tea Station in Fashalam, Rasht, Iran. All the plant materials were selected based on clonal selection method from different tea plantations (open pollinated population) in western part of Guilan province by Tea Research Center, Horticultural Sciences Research Institute, Agricultural Research, Education and Extension Organization (AREEO) of Iran. Then, all selected accessions were collected and cultivated at the station of the Tea Research Center, Lahijan, Guilan province (Latitude 37°15′54′′ N, Longitude 38°45′49′′ E, and − 10 m AMSL). No special permissions were necessary to collect samples. Otherwise, the plant materials used and collected in the study comply with Iran’s guidelines and legislation.

**Table 4:** The name and characteristics of tea accessions studied at the Fashalam tea research station

Row	Accession name	Type	Origin	Yield	Quality
1	100	Chinese	West Guilan	High	Good quality
2	277	Chinese	West Guilan	Medium	Medium quality
3	270	Chinese	West Guilan	Medium	Medium quality
4	74	Chinese	West Guilan	Medium	Medium quality
5	269	Chinese	West Guilan	Medium	Medium quality
6	276	Chinese	West Guilan	High	Good quality
7	399	Chinese	West Guilan	Medium	Medium quality
8	272	Chinese	West Guilan	Medium	Medium quality
9	285	Chinese	West Guilan	High	Good quality
10	278	Chinese	West Guilan	Medium	Medium quality
11	252	Chinese	West Guilan	Medium	Medium quality
12	261	Chinese	West Guilan	Medium	Medium quality
13	280	Chinese	West Guilan	Medium	Medium quality
14	Bazri	Chinese	West Guilan	Medium	Medium quality

All the agronomic operations such as fertilization, pest, diseases, and weeds management were carried out according to the tradition of the area. Irrigation in both experiments was performed until July 22 and after that, irrigation was stopped in the drought stress treatment until August 22 when tea leaves were harvested. Under normal conditions, the irrigation of accessions was carried out every 3 days. Then the leaves were harvested in both conditions and stored in the freezer at − 80 °C for subsequent experiments.

### Regional weather features

The average, standard deviation, and variability of monthly, seasonal and annual rainfall were evaluated for the period under investigation. Also, the proportional contribution of seasonal to annual rainfall was analyzed to understand the rainfall distribution pattern. The annual rainfall was estimated at 1441 mm and the average annual temperature was 16.8 °C, based on the average data of 10 years. Based on the meteorological divisions, this region is part of warm Mediterranean regions with warm summers and mild winters. Although the average annual precipitation of the test area was very high, the distribution of the rainfall throughout the year was generally so low that during the period of tea growth and development, from June to September, the amount of rainfall was not high and it was about zero. Irrigation was suspended for 1 month under drought stress conditions and evaluation of drought stress was performed using gypsum block. Soil moisture content was measured at the field capacity, wilting point in the laboratory and based on block resistance information against volumetric soil moisture, the graph was plotted. Based on block resistance data in different days after stress, it was observed that after 7 days of irrigation interruption, drought stress was gradually applied and severe stress was observed in the field at the end of stopping irrigation.

### Extraction

To extract leaf tea, the harvested leaf of tea accessions in two irrigation conditions was powdered. First, 2 g of the powdered leaves of each tea accession was added to 40 ml of 80% (v/v) methanol [67]. Then, 10 ml of 6 M HCl was added and incubated at 90 °C for 2 h. The mixture was filtered using Whatman filter paper and finally evaporated with a rotary evaporator until completely dry. The dried crude extract was stored at − 20 °C for future studies.

### Measuring the antioxidant activity by DPPH assay

To evaluate the antioxidant activity of the extracts, DPPH-free radicals (1,1-Diphenyl-2-picrylhydrazyl) were measured by using the method of G-C Yen, H-Y Chen and H-H Peng [68]. The different concentrations of the plant dried crude extract dissolved in methanol. Then, the mixture ratio of 1:1 was prepared for DPPH (8 mg/100) solution and tea extracts with different concentrations. After 30 min, at laboratory temperature and darkness, the absorbance of the samples was measured at 517 nm by a spectrophotometer. The percentage of DPPH free radical inhibition was calculated by the following equation:

$$\mathrm{DPPH}\ \mathrm{scavenging}\ \mathrm{effect}\ \left(\%\mathrm{inhibition}\ \mathrm{of}\ \mathrm{DPPH}\ \mathrm{activity}\right)=\frac{\mathrm{Control}\ \mathrm{OD}-\mathrm{Sample}\ \mathrm{OD}\ }{\mathrm{Control}\ \mathrm{OD}}\times 100$$

Where Control OD and Sample OD are the absorbance value of the blank (positive control) and test sample (absorbance of the samples), respectively. To compare the activity of extracts, the concept of IC50 was used. A percent inhibition curve against different concentrations of the sample was drawn and 50% inhibition (IC50) was determined. IC50 is a concentration of extract that eliminates 50% of free radicals. To compare the antioxidant activity of the treatments, Butylated HydroxyToluene (BHT) was used as an industrial antioxidant. Butylated hydroxytoluene (BHT), also known as dibutyl hydroxytoluene, is a lipophilic organic compound, chemically a derivative of phenol, that is useful for its antioxidant properties. BHT is a lab-made chemical that is added to food and is dangerous to human health. It is a chemical with high antioxidant activity and is used to compare the antioxidant activity of natural substances.

### Measurement of phenolic compounds

The total phenolic content of the extract was measured by the Folin–Ciocalteu method [69]. 1 ml of Folin–Ciocalteu reagent and 0.8 ml of sodium carbonate (7.5%) were added to 200 μl of tea extracts. Finally, the absorption of all samples (after storage for 1.5 h at 30 °C and darkness) was determined by spectrophotometer at 650 nm. Also, a series of volumes from 0.2–1 mL of standard solution was made and the standard curve was prepared. Finally, the concentration of phenols in the test sample was calculated using a standard curve and expressed as mg phenols/100 g material.

### Antioxidants gene expression analysis

RNA extraction was performed using Denazist© Total RNA Isolation Kit. To do this, about 100 mg of plant tissue was thoroughly crushed and powdered with liquid nitrogen in a cold Eppendorf tube using a glass rod. Then 1 ml of G1 buffer was added to the microtube and vortexed over 2 periods of 15 s. After incubation in the laboratory for 5 min, the microtubes were centrifuged at 4 °C for 12 min at a speed of 12,000 rpm. The supernatant was transferred to a new microtube and 200 μL of chloroform was added and shaken or vortexed for 15 s. The tube was incubated at laboratory temperature for 3 min and then centrifuged at 4 °C for 12 min at a speed of 12,000 rpm. The supernatant containing the RNA was transferred to a new microtube and half of its volume was added to isopropanol and G2 buffer, respectively. After mixing the contents of the tube and incubating for 10 min at lab temperature, it was centrifuged at 4 °C for 10 min at a speed of 1000 rpm. The supernatant was added cautiously to the residue of 1 mL ethanol (70%), prepared with ribonuclease-free water, and centrifuged again for 5 min at 4 °C at a speed of 10,000 rpm. The supernatant was then removed cautiously and kept for 15 min under the hood at room temperature to dry the precipitate. Then, 30–100 μl of RNase-free water was added to the approximately dried precipitate. The resulting solution was kept in smaller quantities at − 80 °C for subsequent experiments. The cDNA was synthesized using the Thermo Scientific (USA) kit as follows. 1–2 μg of total extracted RNA (treated with DNase and finally reached 10 μl without RNAse) was mixed with 1 μL of Oligo dT and incubated at 75 °C for 5 min. 1 μl of RNase inhibitor (10u / μl), 4 μl of 5X RT buffer, 2 μL of dNTP (10 mM), 2 μL of DTT (0.1 M), and 0.5 μL of Avian Myeloblastosis Virus Reverse Transcriptase enzyme (AMV RT) (10u / μl) were added to the vials, respectively, on ice and spin for a few seconds. The mixture was heated at 42 °C for 60 min (for RT enzyme activity). The reaction was stopped by placing the tubes for 10 min at 70 °C and cooling them on ice.

The sequences of the genes encoding ascorbate peroxidase, catalase, superoxide dismutase, and peroxidase enzymes were extracted from J Li, Y Yang, K Sun, Y Chen, X Chen and X Li [70] (Table 2). Quantitative comparison of gene expression was performed using real-time PCR using ABI (Applied Biosystems step-one) machine. The constructed cDNA was used as a template and qRT - PCR reactions were performed using specific primers for genes encoding antioxidant enzymes and actin as the internal control gene (Table 5). Three replicates were assigned for each reaction. The reaction mixture volume for each sample consisted of 10 μL of SYBR Green mixture, 3 μL of cDNA, 1.8 μL of each of the precursor-specific primers with a concentration of 10 μm, and 2.6 μL of sterile distilled water. The polymerase chain reaction was performed under the following conditions: 10 min at 95 °C and 35 repetitions: 15 s at 95 °C, 15 s at 60 °C, and 45 s at 60 °C for cDNA synthesis. The 2^−△△CT^ method was used for the quantitative analysis of the genes [71].

**Table 5:** Primers used for gene expression analysis

Name	Forward primer(5′-3′)	Reverse primer(5′-3′)	Accession number
*CsSOD*	GATGACGGAACTGCTTGCTT	ATCAGGGTCTGCATGGACAA	AY694187.1
*CsPOD*	GCCACACTTCGCTTATTCTT	AGCCAGGACTACAACATCTC	XM_028207968.1
*CsCAT*	CCTGAACGTGTTGTCCATGC	AACCTCGAGGATCCCTCAG	KR819178.1
*CsAPX*	CAGTTCCCGATGATCTCTTATGC	GCAACAATGTCCTTGTCAGTGAG	DQ442272.1
*Csβ-actin*	GATTCCGTTGCCCTGAAGTCCT	CCTTGCTCATACGGTCTGCGATA	KJ946252.1

### Statistical analysis

Before the simple and combined analysis of variance, assumptions about the normal distribution of distortions and the uniformity of intra-treatment variances were investigated. The mean comparison of traits was performed using the Tukey test. All the analyses were performed using SAS ver. 9.4 [72].

## Data Availability

The data of this study are available from the corresponding author upon request.
